# Endocervical adenocarcinoma of the gastric type: a case report and literature review

**DOI:** 10.3389/fonc.2025.1457253

**Published:** 2025-05-13

**Authors:** Yiting Yang, Xi Zhu, Yayan Zhou, Zihuang Li, Jingqing Liu, Minjie Fang

**Affiliations:** ^1^ Department of Radiation Oncology, Shenzhen People’s Hospital, The Second Clinical Medical College, Jinan University, Shenzhen, China; ^2^ Department of Reproductive Medicine, Shenzhen People’s Hospital, The Second Clinical Medical College, Jinan University, Shenzhen, China; ^3^ Department of Plastic Surgery, Shenzhen People’s Hospital, The Second Clinical Medical College, Jinan University, Shenzhen, China

**Keywords:** gastric-type, endocervical adenocarcinoma, CA19-9, radiotherapy, immunotherapy

## Abstract

Gastric-type endocervical adenocarcinoma (G-EAC) is the most common type of non-human papillomavirus (HPV)-related cervical cancer. Specifically, G-EAC is highly malignant, has a poor prognosis, and is often missed or misdiagnosed, underscoring the need for caution. Herein, we report a case of a patient with G-EAC who underwent surgery, postoperative chemoradiotherapy, targeted therapy, and immunotherapy. Clinical symptoms such as vaginal aqueous secretion and elevated serum carbohydrate antigen-19-9 (CA19-9) levels may aid in diagnosing G-EAC. Of note, in PD-1-negative G-EAC patients, immunotherapy could offer potential benefits. Raising awareness and vigilance regarding this disease, along with developing highly specific antibodies for its early diagnosis, is crucial for improving patient outcomes.

## Introduction

Cervical cancer is the fourth most common malignancy in women worldwide and one of the leading causes of cancer-related deaths among women (1). While most cases are associated with persistent infection by high-risk human papillomavirus (HPV), a small subset is unrelated to HPV infection, primarily consisting of certain types of cervical adenocarcinoma. Gastric-type endocervical adenocarcinoma (G-EAC), the most prevalent form of non-HPV-related cervical cancer, displays features characteristic of gastric-differentiated mucinous adenocarcinoma and shares morphological similarities with pyloric gland epithelium (2). Peutz–Jeghers syndrome (PJS) is a rare autosomal dominant genetic disorder characterized by mucocutaneous pigmentation and gastrointestinal hamartomatous polyposis. Approximately 11% of female PJS patients are diagnosed with G-EAC (3). Compared to usual-type endocervical adenocarcinoma (UEA), G-EAC is highly aggressive, prone to distant metastasis, challenging to treat, and associated with a poor prognosis, with an average overall survival of approximately 2 years. The recurrence rate of G-EAC is significantly higher, at approximately 40% compared to 14.6% for UEA (4), and the 5-year survival rate is markedly lower, at only approximately 32% compared to 70% for UEA (5, 6). The main challenge lies in the atypical clinical manifestations of G-EAC, which make it easy to miss or misdiagnose, leading to treatment delays and severely affecting patient outcomes. To enhance clinicians’ awareness of this disease, we present a case of recurrent G-EAC in a patient whose initial symptom was vaginal watery discharge and who tested negative for HPV. The patient underwent surgery, radiochemotherapy, and immunotherapy.

## Case description

A 60-year-old woman who reached menopause 9 years ago presented to our hospital for treatment in February 2021. Approximately 9 days before hospitalization, she experienced a single episode of vaginal watery secretion that was light red in color and odorless and resolved spontaneously after 1 day. The symptom was not accompanied by dull lower abdominal pain, fever, dizziness, abdominal distension, fatigue, or other discomforts. She underwent a ThinPrep cytologic test (TCT) and HPV genotype test at a local hospital, both of which were negative. However, she underwent hysteroscopy and fractional curettage at the same facility, with pathological findings revealing cervical adenocarcinoma. Subsequently, the patient sought further treatment at our hospital. Her medical history was significant for hypertension, for which she was not on regular medication. She had no other chronic or immune conditions. Her surgical history included a transabdominal bilateral tubal ligation. Obstetric history revealed three pregnancies with no abortions (gravida 3, aborta 0). Notably, both of her sisters were diagnosed with cervical cancer.

**Figure 1:**
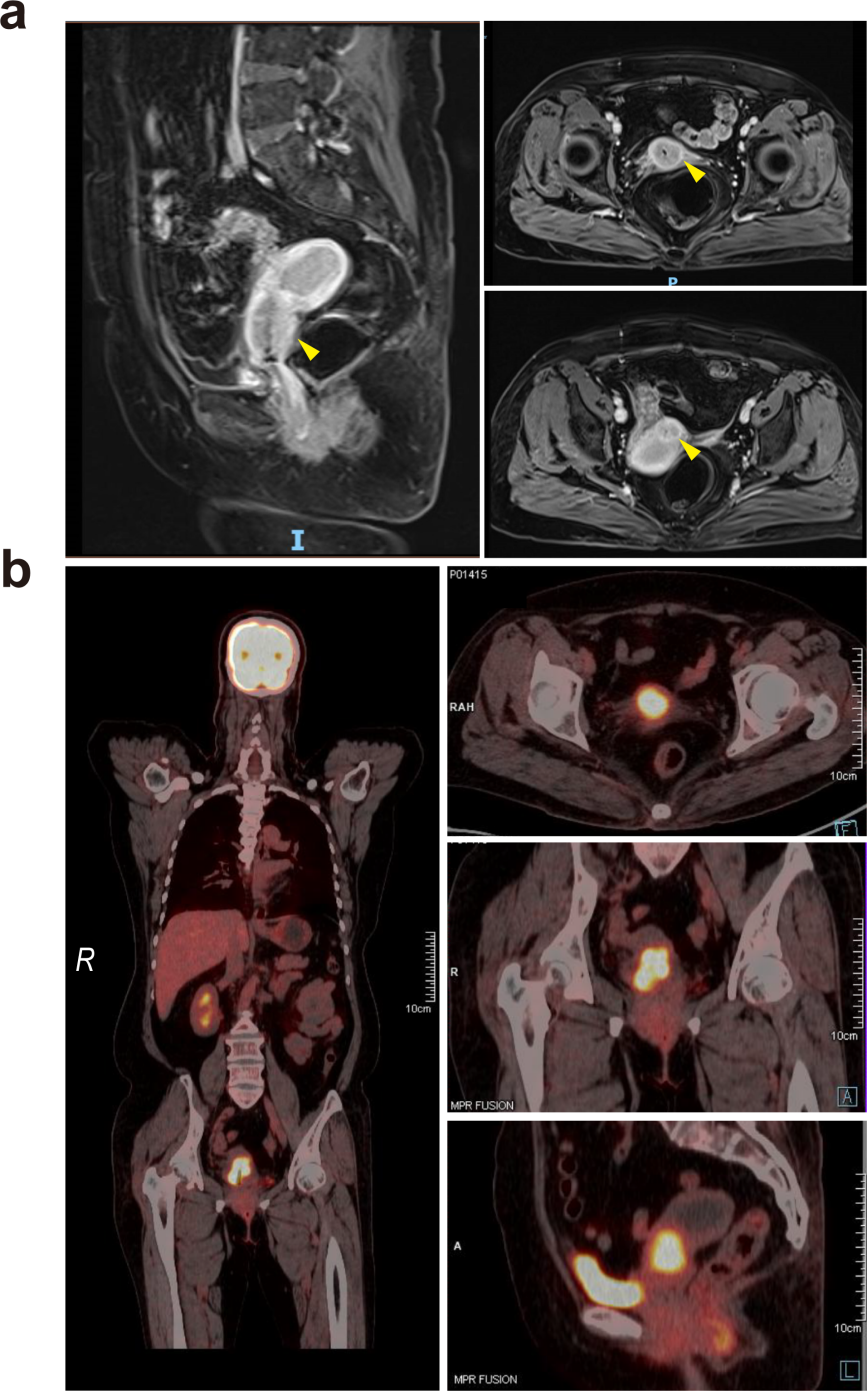
**(a)** Pelvic magnetic resonance imaging revealed a cervical tumor, with yellow arrows indicating the lesion sites. The lesions are primarily located in the upper part of the cervical canal, forming a so-called “barrel-shaped” cervix. **(b)** PET–CT showed hypermetabolic cervical lesions with areas of increased radioactive concentration, measuring approximately 3.5 cm by 3.0 cm, with an Standardized Uptake Value maximum (SUVmax) of 7.8.

**Figure 2:**
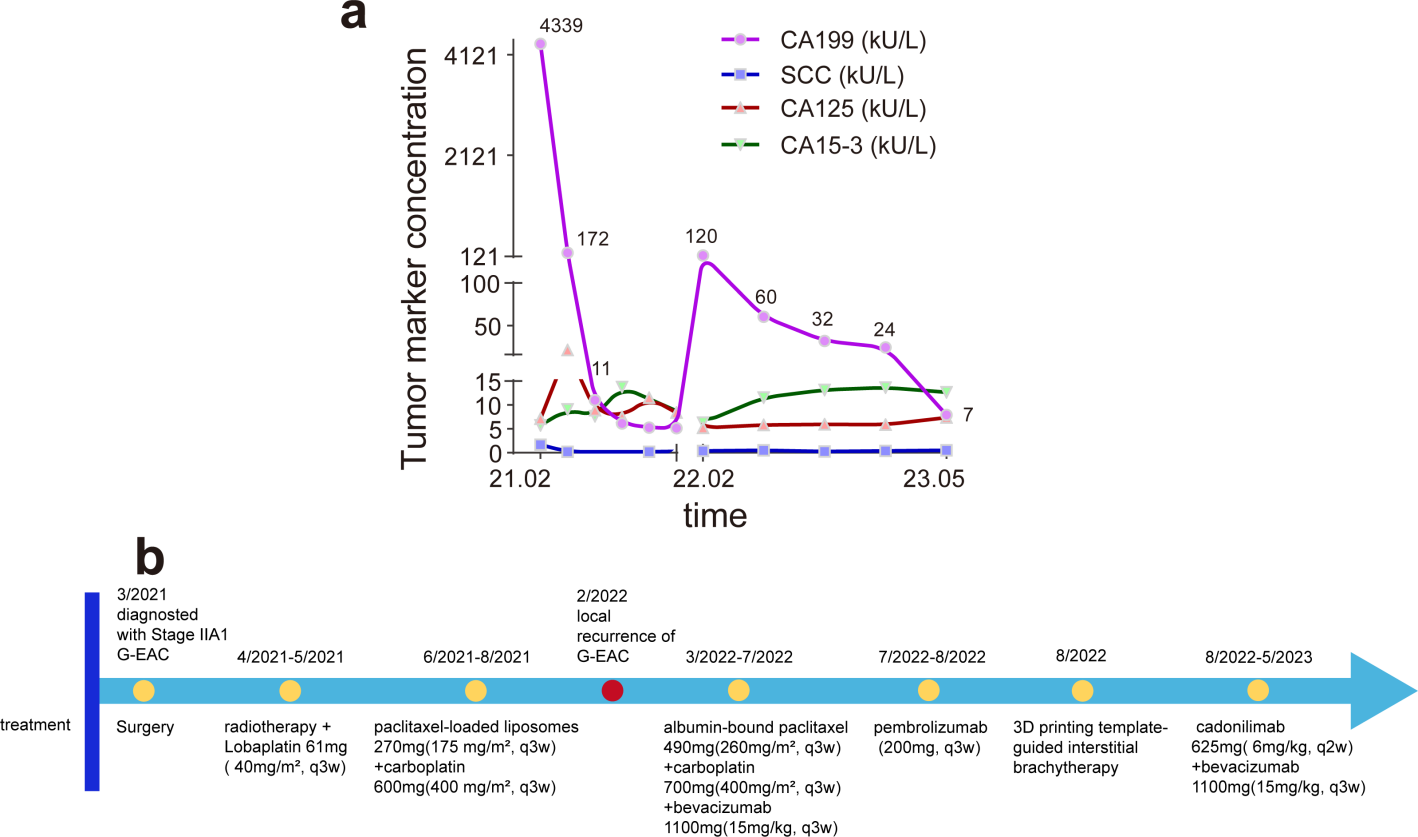
**(a)** The change in tumor markers CA19-9, SCC, CA125, and CA15–3 during treatment and observation. The two CA19–9 peaks occurred preoperatively and during recurrence of gastric-type endocervical adenocarcinoma (G-EAC), suggesting that elevated CA19–9 may indicate the presence or recurrence of G-EAC. CA19-9, carbohydrate antigen 19-9; SCC, squamous cell carcinoma antigen; CA125, cancer antigen 125; CA15-3, cancer antigen 15-3. **(b)** The timeline of major clinical events for the patient since the diagnosis of G-EAC.

**Figure 3:**
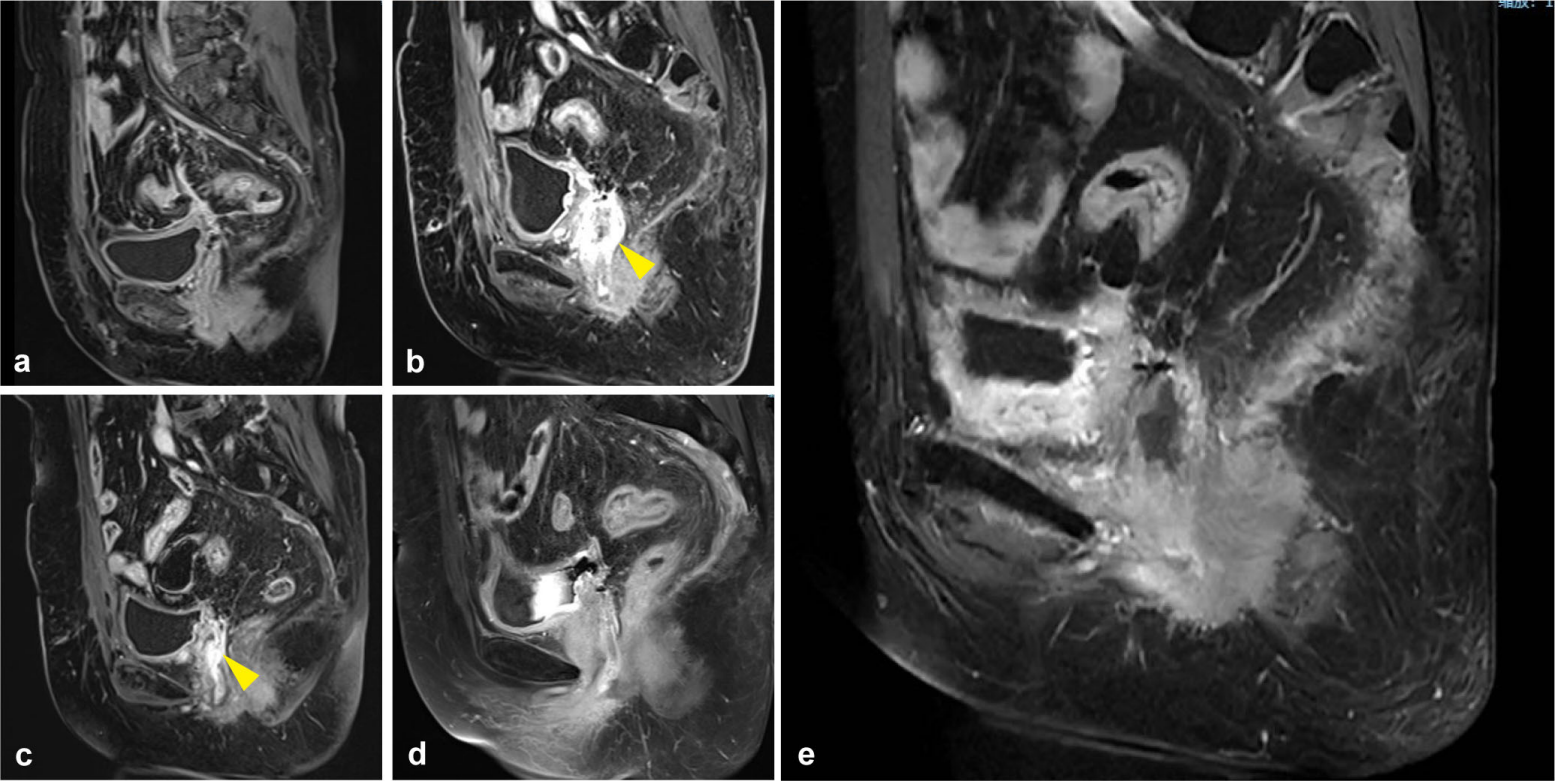
Pelvic MRI during treatment and observation. **(a)** The first whole-abdomen MRI after surgery in April 2021. **(b)** A whole-abdomen MRI in February 2022 revealed a local recurrence of gastric-type endocervical adenocarcinoma (G-EAC), with the yellow arrow pointing to the site of tumor recurrence. The vaginal stump wall shows irregular thickening and enhancement, consistent with tumor recurrence, with possible invasion of the right urethral wall. **(c)** A whole-abdomen MRI in May 2022, with the yellow arrow indicating the tumor site. The vaginal stump wall shows irregular thickening and enhancement, with a reduced extent compared to previous imaging. **(d, e)** As of May 2023, whole-abdomen MRIs indicated no tumor progression.

The patient was initially admitted to the gynecology department. A vagino-recto-abdominal examination revealed a smooth cervical surface with barrel-shaped hyperplasia, no bleeding upon palpation, shallowing of the right vaginal fornix, and slight shortening of the right cardinal and uterosacral ligaments. Pelvic magnetic resonance imaging (MRI) showed circular thickening of the cervical canal mucosa, involvement of the vaginal fornix, and uterine effusion, with no evidence of parametrial or vaginal extension or enlarged pelvic lymph nodes (**Figure 1a**). Positron emission tomography–computed tomography (PET–CT) revealed hypermetabolic cervical lesions measuring approximately 3.5 cm × 3.0 cm invading the lower uterine body and the right vaginal vault, without parametrial or vaginal extension or enlarged pelvic lymph nodes (**Figure 1b**). Tumor markers, including squamous cell carcinoma (SCC) antigen, carbohydrate antigens 153 (CA153) and 125 (CA125), carcinoembryonic antigen (CEA), and alpha-fetoprotein (AFP), were all within normal ranges. However, carbohydrate antigen 19-9 (CA19-9) was significantly elevated at 4,339.00 U/mL (**Figure 2a**). The patient was ultimately diagnosed with cervical cancer, classified as International Federation of Gynecology and Obstetrics (FIGO 2018) stage IIA1. In March 2021, she underwent radical hysterectomy, bilateral adnexectomy, and pelvic lymphadenectomy. Pathological examination revealed poorly differentiated invasive cervical adenocarcinoma of the gastric type (non-HPV-related). The tumor measured approximately 3.5 cm × 1 cm × 0.5 cm and did not invade the vagina. The lesion infiltrated the entire thickness of the cervical muscle wall, with lymphovascular space invasion observed. Surgical margins of the vagina, vaginal fornix, bilateral ovaries, and parametrium were clean. None of the 30 examined pelvic lymph nodes showed evidence of metastasis or invasion. Immunohistochemical analysis indicated the following results: ER(−), PR(−), P16(−), CEA(−), CK7(+), WT-1(−), p53 (wild type, 85%), PAX-8(−), MUC6(−), Ki67 (30%), PD-1(−), and PD-L1 (Combined Positive Score (CPS) < 1). A follow-up MRI scan was performed in April 2021, post-surgery (**Figure 3a**). The patient underwent pelvic radiotherapy in April 2021 using Volumetric Modulated Arc Therapy (VMAT) technology. The treatment included a total dose of 48.6 Gy delivered in fractions of 1.8 Gy over 5 weeks. Concurrently, she received two cycles of 3-week chemotherapy with lobaplatin (61 mg; 40 mg/m^2^, q3w) without experiencing hematologic toxicity. Following radiotherapy, the patient underwent four cycles of chemotherapy with paclitaxel-loaded liposomes (270 mg; 175 mg/m^2^, q3w) and carboplatin (600 mg; 400 mg/m^2^, q3w) from June 2021 to August 2021. At the conclusion of the planned treatment, the patient achieved complete clinical remission of the disease. However, her CA19-9 levels reduced significantly to 5.15 U/mL. She was subsequently monitored through periodic follow-up evaluations.

**Figure 4:**
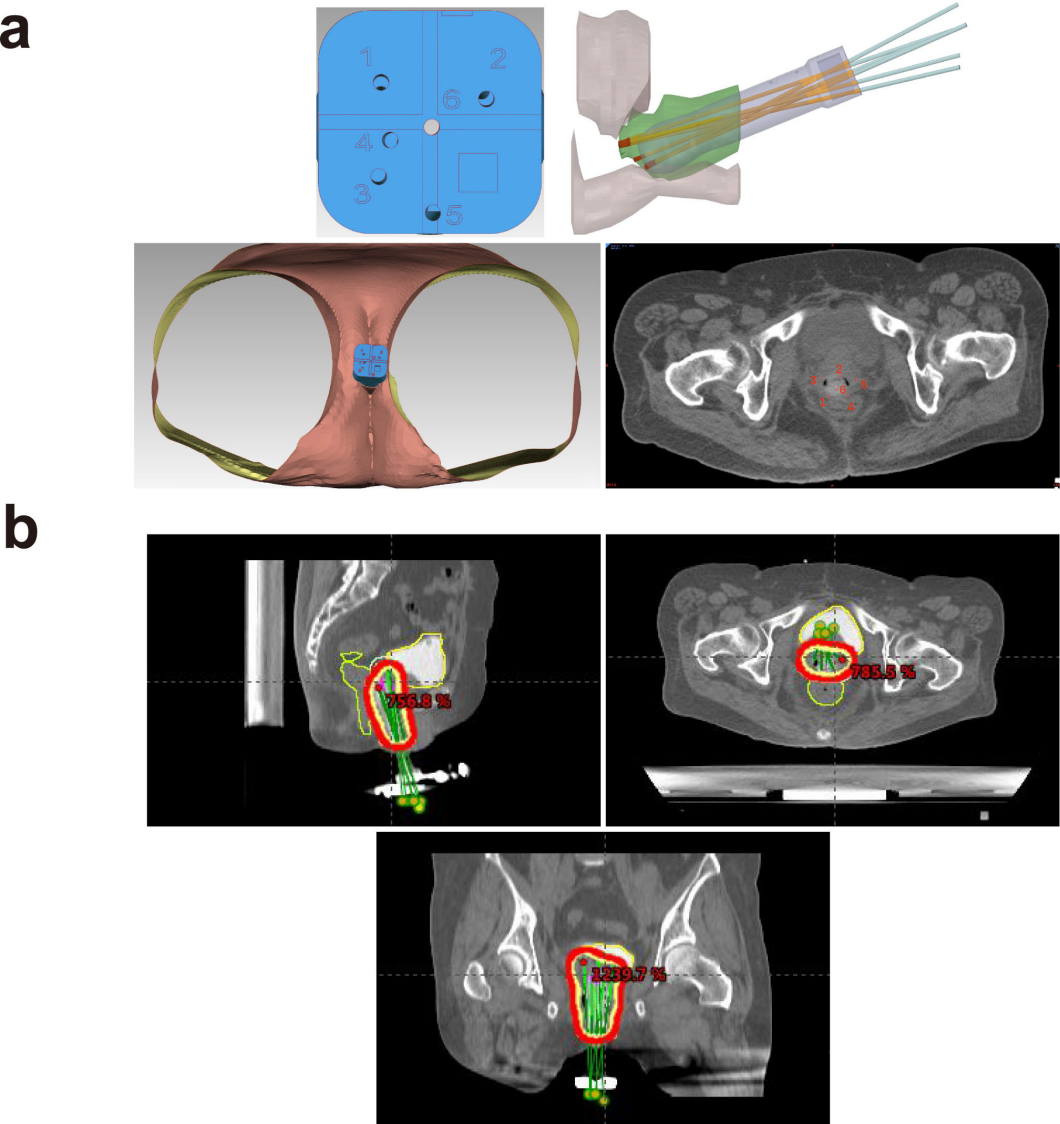
**(a)** Three-dimensional printing coplanar template (3D-PCT)-assisted brachytherapy reconstruction visualization for recurrent cervical cancer. **(b)** Region of 3D printing template-guided interstitial brachytherapy (ISBT) using a ^192^Ir source.

In February 2022, the patient presented with vaginal discharge that was yellow in color and contained a slight amount of blood. A positive acetowhitening test result was observed during vaginal colposcopy, and a biopsy was performed. Pathological examination confirmed the presence of cervical adenocarcinoma. Immunohistochemical analysis revealed that the tumor cells were PD-1(−) and PD-L1 (22C3, CPS < 1). MRI of the pelvis indicated uneven thickening and enhancement of the vaginal stump, consistent with tumor recurrence (**Figure 3b**). PET–CT revealed nodular abnormal radioactive concentration in the vaginal stump measuring approximately 4.3 cm × 2.5 cm × 3.8 cm. No lymph node metastasis was observed in the bilateral iliac vessels or pelvic cavity. CA19-9 levels were significantly elevated to 120.9 U/mL, while other tumor markers remained within normal ranges (**Figure 2a**). The patient was diagnosed with a recurrence of G-EAC after radiotherapy. From March 2022 to July 2022, she underwent six cycles of treatment with albumin-bound paclitaxel (490 mg; 260 mg/m^2^, q3w), carboplatin (700 mg; 400 mg/m^2^, q3w), and bevacizumab (1,100 mg; 15 mg/kg, q3w) as maintenance therapy. Pathological examination showed PD-L1 negativity (CPS < 1), but the patient requested treatment with pembrolizumab. She received two cycles of pembrolizumab (200 mg; q3w) in July and August 2022. Meanwhile, cadonilimab is a bispecific programmed cell death protein-1 (PD-1)/cytotoxic T-lymphocyte antigen-4 (CTLA-4) antibody, and it has been shown in clinical investigations that patients with cervical cancer can benefit from cadonilimab monotherapy, regardless of their PD-L1 expression status (7). Based on this, the decision was made to switch maintenance therapy from pembrolizumab to cadonilimab (625 mg; 6 mg/kg, q2w). In August 2022, 3D printing template-guided interstitial brachytherapy (ISBT) with a ^192^Ir source was administered at the conclusion of chemotherapy. The single dose of interstitial brachytherapy was 6 Gy, administered weekly for four fractions (**Figure 4**). The patient continued to receive bevacizumab combined with cadonilimab treatment. In November 2022, a follow-up PET–CT showed a mildly abnormal radioactive concentration in the vaginal stump, measuring approximately 2.4 cm × 1.4 cm, with a significant reduction in the size of the lesion compared to that in previous scans. No obvious lymph node metastasis was observed in the bilateral iliac vessels or pelvic cavity. Throughout this period, the patient underwent regular follow-up whole-abdomen MRI scans (**Figures 3c-e**), with no disease progression noted. She has been undergoing follow-up examinations at another hospital since May 2023 (for over 1 year). During treatment, no grade ≥ 3 immune-related adverse events (irAEs) or liver toxicity were reported. Leukopenia (grade 2) was managed with granulocyte colony-stimulating factor. MRI scans were performed every 3 months, and regular monitoring of blood routine, liver function, kidney function, electrolyte levels, thyroid function, and pituitary function was conducted. A timeline of the major clinical events since the diagnosis of G-EAC is shown in **Figure 2b**.

## Discussion

G-EAC is the most common type of non-HPV-related cervical cancer (2). The occurrence of G-EAC is unrelated to high-risk HPV infection (8), and the biopsy-positive rate is lower compared to that of cervical squamous cell carcinoma. The clinical manifestations of G-EAC are highly atypical, and its biological characteristics are notably malignant. Compared to usual-type endocervical adenocarcinoma, G-EAC is more significantly associated with features such as bulky mass, deep stromal invasion, lymphovascular invasion, parametrial invasion, ovarian metastasis, and positive ascitic fluid cytology (4). These factors contribute to its high susceptibility to misdiagnosis, leading to delays in treatment and poorer prognosis for patients.

The identification and management of precursor lesions are crucial for the prevention and early detection of cancer. It is reported that 99.7% of cervical cancer cases can be attributed to high-risk HPV (hrHPV) infection (9), making HPV testing one of the key methods for primary screening of cervical cancer (10). However, the occurrence of G-EAC is not related to high-risk HPV infection. The International Endocervical Adenocarcinoma Criteria and Classification (IECC) utilizes the advanced cell-based RNAscope diagnostic system for HPV *in situ* hybridization testing, and all G-EAC cases detected by this method are HPV-negative (8). Therefore, HPV detection has limited value in the diagnosis of G-EAC. The typical clinical manifestation of G-EAC is vaginal watery discharge, while irregular vaginal bleeding is less commonly observed compared to that in cervical squamous cell carcinoma. In G-EAC, the external cervical orifice often appears smooth or eroded, and the cervix may be enlarged without visible external lesions. The lesions are typically located in the upper part of the cervical canal, resulting in a so-called “barrel-shaped” cervix (4, 11, 12). In such cases, the likelihood of referring patients for colposcopic examination is low, and the diagnostic rate of cervical biopsy is generally low, approximately 30% to 40%. Additional procedures, such as multiple cervical biopsies, diagnostic curettage, or cervical conization, may increase the diagnostic rate to approximately 50% (13). In our case, the patient presented with vaginal discharge, a smooth cervical surface with barrel-shaped hyperplasia, and a negative HPV examination, all of which underscore the challenges of early diagnosis of G-EAC. Thus, for patients presenting with vaginal watery discharge and negative HPV results, the possibility of G-EAC should be considered. A combination of cervical cytology, MRI, and cervical biopsy are effective methods for the early detection and diagnosis of G-EAC.

Tumor markers are widely used in clinical practice to assess therapeutic efficacy, detect recurrence, and predict the prognosis of known cancers. In the case of G-EAC, elevated tumor markers may be observed. Although SCC antigen is typically considered a representative tumor marker for cervical cancer, other markers, such as CA19–9 and CA125, also play an important role in the diagnosis of gynecological tumors, particularly ovarian cancer (14, 15). Nonetheless, these findings are atypical for common cervical cancer. Herein, we observed that CA19–9 levels were significantly elevated both at the initial diagnosis and upon recurrence, maintaining substantial diagnostic value for G-EAC. In contrast, SCC did not show significant changes in CA19–9 levels. It has been reported that more than half of G-EAC patients exhibit elevated CA19–9 levels (16). We recommend that clinicians consider the possibility of G-EAC when encountering patients with elevated CA19–9 levels. CA19–9 may also be elevated in cases of gastrointestinal tract malignancies, including those of the pancreas, colorectum, and biliary tract (17). G-EAC shares several genetic characteristics with both gastric and intestinal adenocarcinomas. TP53 mutations are common in both G-EAC and gastrointestinal adenocarcinomas. These are followed by STK11, HLA-B, PTPRS, BRCA2, Snail, and TWIST1, among others. Furthermore, mutations in KMT2D, ERBB2/3, and RNF43 are also observed in both G-EAC and gastric adenocarcinoma (2, 18). These mutated genes are primarily involved in signal transduction, DNA damage repair, and epithelial–mesenchymal transition (EMT) (2, 19–21). The loss of TP53 function disrupts critical cell cycle regulation and reduces DNA damage repair efficiency, ultimately increasing tumor cell resistance to chemotherapy and radiation treatment. ERBB2 mutations lead to the constitutive activation of the HER2 pathway, driving tumor proliferation and survival (22). Although HER2 amplification may confer sensitivity to trastuzumab, concurrent PIK3CA mutations can induce resistance via the PI3K/AKT pathway (23). Mutations related to EMT may also contribute to tumor dissemination and chemoresistance in G-EAC. Snail, a central EMT regulator, promotes metastasis by suppressing epithelial markers like E-cadherin, thereby enhancing invasiveness and therapy resistance, leading to poor prognosis (24). Importantly, these shared genetic features could provide valuable insights into the unique aspects of G-EAC.

Due to the rarity of this pathological type, there is currently no standard treatment for G-EAC. Treatment should be tailored based on the guidelines for usual-type endocervical adenocarcinomas and cervical squamous cell carcinoma. Nevertheless, the clinicopathological features and prognosis of G-EAC differ significantly from those of other subtypes. G-EAC is characterized by a high prevalence of TP53 mutations, which result in poor sensitivity to radiotherapy and chemotherapy, increased drug resistance, and a worse prognosis (2, 4). As a result, treating G-EAC is highly challenging, and further treatment experience is still needed. Surgery was selected as the primary treatment due to the tumor stage and clinical presentation. The patient had negative surgical margins, met the Sedlis criteria, and then received postoperative pelvic external beam radiation therapy (EBRT) with concurrent platinum-containing chemotherapy (lobaplatin). Despite this, the patient experienced recurrence in the vaginal stump within 1 year. Therefore, even though postoperative pathology indicated negative lymph nodes, negative surgical margins, and negative parametrium, we still recommend adding vaginal brachytherapy after completing pelvic EBRT in G-EAC patients.

In recent years, significant progress has been made in the field of immunotherapy for cervical cancer. Clinical investigations of cadonilimab have shown that patients can benefit from cadonilimab monotherapy, regardless of their PD-L1 expression levels (25). Here, despite the patient being PD-1-negative, we decided to transition the maintenance therapy from pembrolizumab to cadonilimab, resulting in progression-free survival for over 1 year. Therefore, for recurrent PD-1-negative G-EAC patients, immune checkpoint inhibitor treatment may be considered after obtaining informed consent.

## Conclusions

G-EAC is a rare form of cervical mucinous adenocarcinoma that exhibits highly malignant biological behavior, characterized by strong invasiveness, a tendency to metastasize, drug resistance, and poor prognosis. G-EAC is typically negative for high-risk HPV and presents with non-specific symptoms, most commonly vaginal discharge. The cervix is usually enlarged without an obvious mass. Elevated serum CA19–9 may be observed, and MRI often demonstrates the characteristic “cosmos pattern”. Combined with previous studies, we think that CA19–9 may be considered a potential reference biomarker for monitoring therapeutic outcomes and recurrence in G-EAC. Through our case, the use of cadonilimab may offer potential benefits in the treatment of G-EAC and warrants further investigation. Identifying genetic and molecular alterations and developing biomarkers to identify therapeutic targets will be essential for enabling early diagnosis and precision therapies.

## Data Availability

The original contributions presented in the study are included in the article/supplementary material. Further inquiries can be directed to the corresponding author.

## References

[1] Kirthana S Dorothy AM Zheng QT Demisew A Mazvita M Andrew KN. No woman left behind: achieving cervical cancer elimination among women living with HIV. Lancet HIV. (2023) 10(6):e412–20. doi: 10.1016/S2352-3018(23)00082-6 37182539

[2] Eunhyang P Sang Wun K Sunghoon K Hyun-Soo K Jung-Yun L Young Tae K. Genetic characteristics of gastric-type mucinous carcinoma of the uterine cervix. Mod Pathol. (2020) 34(3):637–46. doi: 10.1038/s41379-020-0614-0 32641744

[3] Kim Y Kim EY Kim TJ Lim KT Lee KH Chun Y. A rare case of gastric-type mucinous adenocarcinoma in a woman with Peutz-Jeghers syndrome. Obstetrics gynecol Sci. (2019) 62:474–7. doi: 10.5468/ogs.2019.62.6.474 PMC6856476 31777745

[4] Shin N Yoshiki M Hideki T Nobuo Y Toyomi S Motoaki S. Analysis of gastric-type mucinous carcinoma of the uterine cervix - An aggressive tumor with a poor prognosis: A multi-institutional study. Gynecol Oncol. (2019) 153(1):13–19. doi: 10.1016/j.ygyno.2019.01.022 30709650

[5] Atsumi K Yoshiki M Tamotsu S Satoshi Y Yasuki K Masaharu I. Gastric morphology and immunophenotype predict poor outcome in mucinous adenocarcinoma of the uterine cervix. Am J Surg Pathol. (2007) 31(5):664–72. doi: 10.1097/01.pas.0000213434.91868.b0 17460448

[6] Gulisa T Kay JP. Cervical glandular neoplasia: classification and staging. Surg Pathol Clin. (2019) 12(2):281–313. doi: 10.1016/j.path.2019.01.002 31097105

[7] Wu X Sun Y Yang H Wang J Lou H Li D. Cadonilimab plus platinum-based chemotherapy with or without bevacizumab as first-line treatment for persistent, recurrent, or metastatic cervical cancer (COMPASSION-16): a randomised, double-blind, placebo-controlled phase 3 trial in China. Lancet. (2024) 404:1668–76. doi: 10.1016/s0140-6736(24)02135-4 39426385

[8] Simona S Iulia B Lien H Prusha P Cristina T Anna P. International endocervical adenocarcinoma criteria and classification (IECC): A new pathogenetic classification for invasive adenocarcinomas of the endocervix. Am J Surg Pathol. (2017) 42(2):214–26. doi: 10.1097/PAS.0000000000000986 PMC5762258 29135516

[9] Paul AC Anjua J Ana O Lynette D. Cervical cancer. Lancet. (2019) 393(10167):169–82. doi: 10.1016/S0140-6736(18)32470-X 30638582

[10] Joseph ET James B Jennifer B François C Máire AD Alex F. Introduction of molecular HPV testing as the primary technology in cervical cancer screening: Acting on evidence to change the current paradigm. Prev Med. (2017) 98:5-14. doi: 10.1016/j.ypmed.2016.11.029 28279264

[11] Edyta CP Kay JP Takako K Xun Z Wen C David J. Gastric-type adenocarcinoma of the cervix: tumor with wide range of histologic appearances. Adv Anat Pathol. (2018) 26(1):1–12. doi: 10.1097/PAP.0000000000000216 30234500

[12] Karen LT Glenn McCluggage W. The developing spectrum of gastric-type cervical glandular lesions. Pathology. (2017) 50(2):122–33. doi: 10.1016/j.pathol.2017.09.009 29233547

[13] Guiling L Wei J Suiqi G Congjian X. Minimal deviation adenocarcinoma of the uterine cervix. Int J Gynaecol Obstet. (2010) 110(2):89–92. doi: 10.1016/j.ijgo.2010.03.016 20451906

[14] Francesk M Nikoleta O Levan T Elias L Ioannis K. A giant ovarian mucinous tumor in a 58-year-old postmenopausal patient with persistent abdominal pain and high serum levels of CA 19-9. Pan Afr Med J. (2020) 37:76. doi: 10.11604/pamj.2020.37.76.25932 33244339 PMC7680227

[15] Francesk M Panagiotis T Ioannis M. A giant ovarian mass in a 68-year-old female with persistent abdominal pain and elevated serum CA-125 level. Prz Menopauzalny. (2020) 19(2):108–10. doi: 10.5114/pm.2020.97870 PMC7422285 32802022

[16] Ayano N Ken Y Sachiko M Ryusuke M Kaoru A Junzo H. Mucinous adenocarcinoma, gastric type of the uterine cervix: clinical features and HER2 amplification. Med Mol Morphol. (2018) 52(1):52–59. doi: 10.1007/s00795-018-0202-2 29992451

[17] Lin D Shikong G Hong L Xianghui Y Yang S Haichuan S. CA125, CEA, CA19-9, and heteroploid cells in ascites fluid may help diagnose peritoneal carcinomatosis in patients with gastrointestinal and ovarian Malignancies. Cancer Manage Res. (2020) 12:10479–89. doi: 10.2147/CMAR.S271596 PMC7588672 33122947

[18] Shin N Koji M Hiroki N Kenta M Yoshiki M Nobuo Y. Analysis of postoperative adjuvant therapy in 102 patients with gastric-type mucinous carcinoma of the uterine cervix: A multi-institutional study. Eur J Surg Oncol. (2022) 48(9):2039–44. doi: 10.1016/j.ejso.2022.03.007 35354541

[19] Jinkyoung K Hoiseon J Youngseok L Chungyeul K Hankyeom K Aeree K. HRG-β1-driven ErbB3 signaling induces epithelial-mesenchymal transition in breast cancer cells. BMC Cancer. (2013) 13:383. doi: 10.1186/1471-2407-13-383 23937725 PMC3750857

[20] Kristiina R Aurora T Riku K Niko V Anna K Roosa-Maria P. Genetic and epigenetic characteristics of inflammatory bowel disease-associated colorectal cancer. Gastroenterology. (2021) 161(2):592–607. doi: 10.1053/j.gastro.2021.04.042 33930428

[21] Robert M Daniela S Carla M Wei QL Roger E-F Roser P. Molecular classification and therapeutic targets in extrahepatic cholangiocarcinoma. J Hepatol. (2020) 73(2):315–27. doi: 10.1016/j.jhep.2020.03.008 PMC8418904 32173382

[22] Nishio S. Current status and molecular biology of human papillomavirus-independent gastric-type adenocarcinoma of the cervix. J Obstet Gynaecol Res. (2023) 49:1106–13. doi: 10.1111/jog.15578 36759334

[23] Ojesina AI Lichtenstein L Freeman SS Pedamallu CS Imaz-Rosshandler I Pugh TJ. Landscape of genomic alterations in cervical carcinomas. Nature. (2014) 506:371–5. doi: 10.1038/nature12881 PMC4161954 24390348

[24] Park E Kim SW Kim S Kim HS Lee JY Kim YT. Genetic characteristics of gastric-type mucinous carcinoma of the uterine cervix. Mod Pathol. (2021) 34:637–46. doi: 10.1038/s41379-020-0614-0 32641744

[25] Hanmei L Hongbing C Xin H Guiling L Li W Fei L. Cadonilimab combined with chemotherapy with or without bevacizumab as first-line treatment in recurrent or metastatic cervical cancer (COMPASSION-13): A phase 2 study. Clin Cancer Res. (2024) 30(8):1501–8. doi: 10.1158/1078-0432.CCR-23-3162 PMC11016896 38372727

